# Mechanisms of cyst formation in metastatic lymph nodes of head and neck squamous cell carcinoma

**DOI:** 10.1186/1746-1596-7-6

**Published:** 2012-01-16

**Authors:** Sepideh Mokhtari

**Affiliations:** 1 Department of Oral and Maxillofacial Pathology, Shahid Beheshti University of Medical Sciences, Velenjak street, Tehran, Iran

**Keywords:** cyst, lymph node, metastases, primary tumor, squamous cell carcinoma

## Abstract

Cystic change in metastatic lymph nodes occurs in certain types of tumors and mostly in squamous cell carcinoma of the head and neck. In the majority of cases, psuedocystic change is the mechanism of cyst formation. However, sometimes a true cyst cavity is formed. This occurrence is unexplained and some theories are introduced to explain it. In this paper, related articles and introduced concepts are reviewed and the best conclusions of present hypotheses are provided. Cystic SCC in cervical lymph node is now considered as a typical presentation of metastatic SCC arising in the oro/nasopharynx. True cystic cavities have eosinophilic fluid content and present active transport mechanism across the epithelium; Cytokeratin7 is also expressed in the lining of these cysts, which is an accepted marker of ductal differentiation. These are all strong evidences that show salivary gland type cells are present among tumor cells. In fact, some squamous cell carcinomas, especially those arising in Waldeyer's ring, originate from minor salivary glands. The other probability is that these tumors are cancers of transitional type and arise from transformed keratinocytes, which have intrinsic property for cyst formation. These malignant cells in lymph nodes, rather than primary sites, found the opportunity to express their parental property.

**Virtual slides:**

The virtual slide(s) for this article can be found here:

http://www.diagnosticpathology.diagnomx.eu/vs/6838476096250792.

## Introduction

Cystic change in metastatic lymph nodes occurs in certain types of tumors and it is an unexplained, site-specific phenomenon that mostly happens in the lymph nodes of head and neck region. It is also found with decreasing frequency in the inguinal, axillary and supraclavicular regions. The reported primary tumors are most commonly squamous cell carcinoma (SCC) and thyroid papillary carcinoma. As well, cystic metastases rarely have been encountered in other tumors such as serous papillary carcinoma of the ovary or endometrium and malignant melanoma [1].

**Figure 1:**
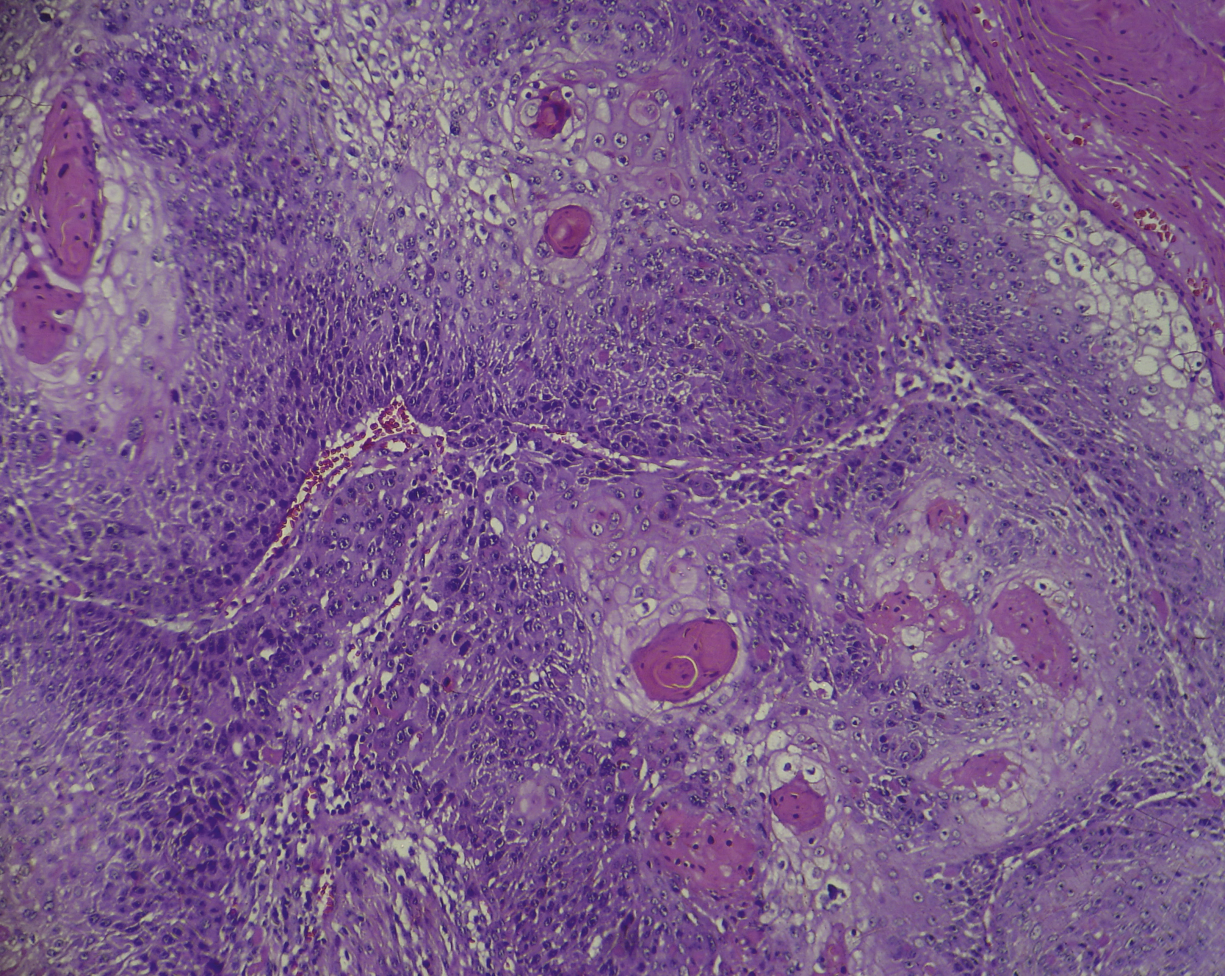
**Primary tumor; solid tumor nests in a case of head and neck squamous cell carcinoma in primary location**.

**Figure 2:**
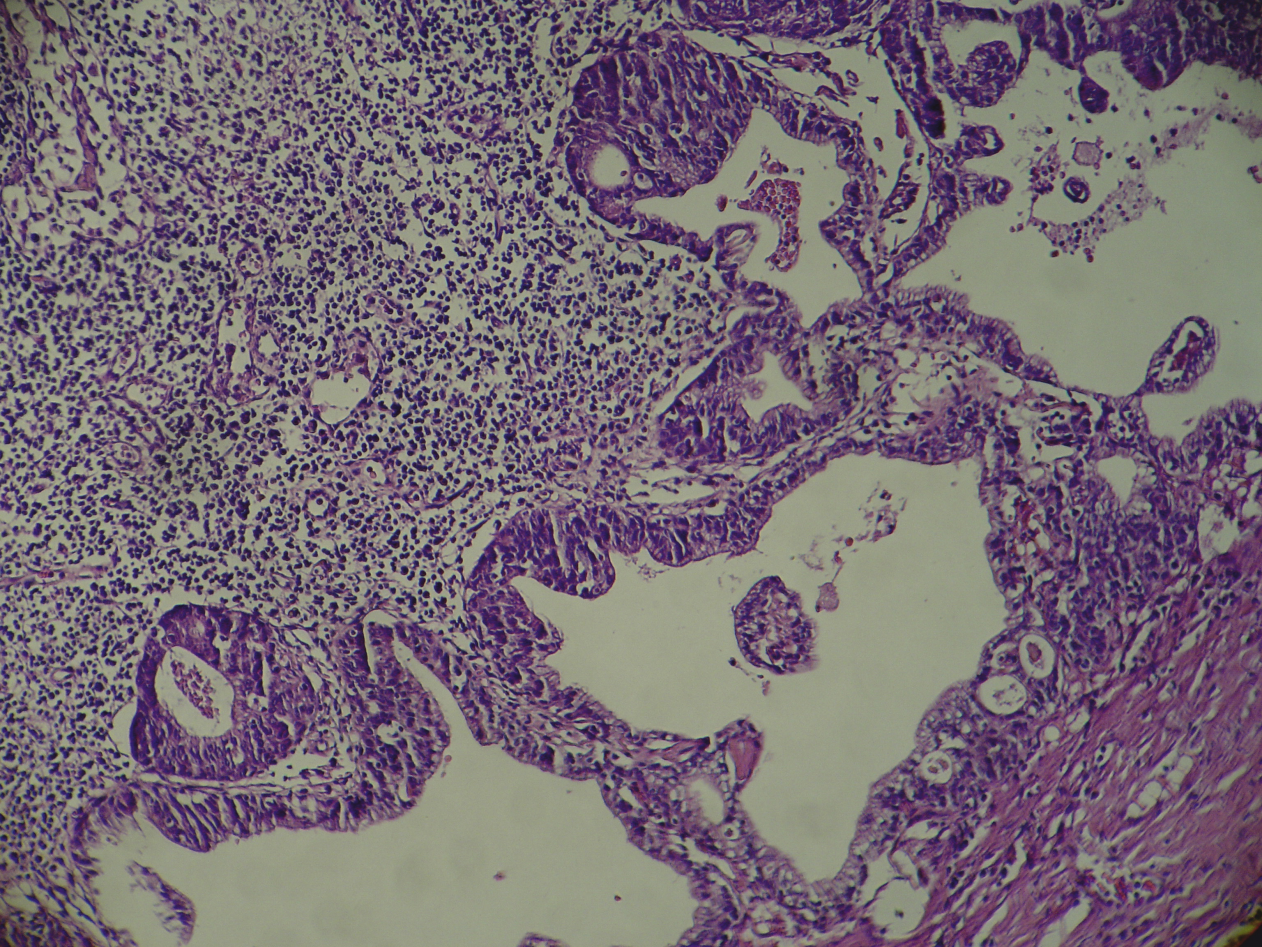
**Metastatic lymph node; cystic structures in the metastatic cervical lymph node of the same case**.

In case of a cystic nodal metastasis of SCC, the primary tumor is solid [Figure 1] however metastatic lymph node presents one or multiple cystic structures [Figure 2]. In these cases, 72-90% of primary tumors, when detected, are located in Waldeyer's ring (base of the tongue, palatine tonsils, and nasopharynx) [2,3]. Larynx, hard palate, thyroid gland, salivary glands, sinuses [2,4], lung, uterine cervix [5] and esophagus [6] are the other probable sites but reported cases are rare.

Although psuedocystic change is the mechanism of cyst formation in the majority of cases [7], sometimes a true cystic cavity is formed. This occurrence is not well investigated; however, some theories are introduced which form the basis of this article.

## Methods

Here, a thorough review on the literature is provided and the main concepts about mechanisms of cyst formation in metastatic lymph nodes of squamous cell carcinoma are summarized. There are some differences between real cysts and pseudocysts, which are presented in table 1. Primary site and metastatic lymph node are different environments and have different effects on malignant cells; these environmental conditions are also demonstrated in table 2. As well, different genetic content of primary and metastatic tumors is considered and probable associations with cyst formation are discussed.

**Table 1:** Differences between real cysts and pseudocysts in metastatic lymph nodes

Cavity	prevalence	Lumen content	CK7 Expression	Mechanism of formation
**Pseudocysts**	Majority of cases	keratin and cellular debris	Negative	Degradation of keratin and cellular debris/Sudden blockage of lymphatic fluid flow

**True cysts**	Few cases	eosinophilic fluid	Positive	Origination from transformed keratinocytes/ducts of submucosal salivary glands

**Table 2:** The effect of environment on malignant cells

Environment	Environmental condition for malignant cells	Proliferation rate	Acquisition of mutations	Tumor presentation
**Primary site**	Suitable environment	High	High	Low expression of intrinsic properties

**Lymph node**	Strong immunologic host response, Harsh environment	Low	Low	Silent presentation of tumor and expression of intrinsic properties

## Discussion

It is possible that a cystic carcinoma in a lymph node be a primary tumor or have origination from a benign cyst already present in the lymph node. However, these probabilities seem to be a hypothetical entity, or an extremely rare event [7]. Cystic SCC in metastatic cervical lymph nodes is now considered as a typical presentation of SCC arising in the oro/nasopharynx [7]. It has been suggested that this phenomenon is secondary to pseudocystic change and results from spontaneous degradation of keratin and cellular debris within the carcinomatous lymph node deposit [5]. Probably cyst formation in these cases could also be related to the sudden blockage of lymphatic flow passing through a node that has metastatic colony. This lymphatic fluid fills a potential space, which have tumor cells in periphery [8].

As well, real cystic metastases are cavities lined by malignant cells and the periphery is occupied with lymphoid elements [9]. The mechanism of these true cysts remains elusive. How could this occurrence be explained? Why are not these features demonstrated in the primary sites so clearly? Is there any association between lymphatic environment and cyst formation? Could it be related to the differences in genetic content of primary and metastatic tumor? Answering to all these questions needs through investigations in different histopathological and genetic aspects of the subject. Here, some discussions are provided:

### Human papilloma virus and cystic metastases of SCC

Some studies have found associations between cystic lymph node metastases and human papilloma virus (HPV) [2]. It has been noted that nodal metastases from HPV-related SCCs of the upper aerodigestive tract are prone to be cystic [10].

### Transitional type of SCC and cystic metastases

The incidence of cystic lymph node arising from SCC of Waldeyer's ring has been reported to be as high as 37% to 62% of those with nodal metastasis [5,7]. Thompson and Heffner [2] found that primary tumors of tongue base that show cystic nodal metastases are not a usual SCC. In fact, they are cancers of transitional type that occur at the base of tongue. The unique feature of this type is its propensity for moderately large cystic metastases in the cervical lymph nodes [2]. Moreover, cystic and microcystic spaces are often present in primary squamous cell carcinoma of Waldeyer's ring. Therefore, cyst formation is considered as an intrinsic property of malignant keratinocytes of these sites. These transformed keratinocytes when metastasize, show histological patterns of their parent cells [7].

The lumen of pseudocysts is filled with cellular debris and transport mechanism of any fluid into the lumen is passive. On the other hand, the lumen of true cysts has eosinophilic fluid content. This implicates an osmotic/secretory or an active transport mechanism across the epithelium as seen in salivary gland epithelium and it may happen through displaced salivary gland cells [7]. Regauer et al [11] analyzed the cytokeratin7 (CK7) expression, as an accepted marker for ductal differentiation, in primary and metastatic carcinomas of the Waldeyer's ring. CK7 was expressed in submucosal minor salivary gland acini and ducts but not in the squamous surface epithelium of Waldeyer's ring. They found that CK7-positive carcinomas produced CK7-positive cystic nodal metastases. It seems that some carcinomas occurring in Waldeyer's ring area originate from excretory ducts of submucosal minor salivary glands, express CK7, and produce CK7-positive cystic nodal metastases [11].

As a conclusion, one of the followings can explain the phenomenon of real cyst formation in metastatic lymph nodes:

1. The presence of malignant salivary gland type cells that metastasize and express their parental property in lymph nodes (based on Regauer et al. study [11])

2. Transformed keratinocytes that have intrinsic properties for cyst formation become malignant and produce a transitional type of squamous cell carcinoma (based on Thompson et al. study [2]).

What is evident is that neither a usual SCC nor all keratinocytes can produce true cysts in lymph node metastases.

### Lymphatic environment effect on cyst formation

To evaluate the effect of lymphatic environment on tumoral cells, the following issues should be considered:

1. Lymphocytes around a primary SCC are responsible for silent presentation of a tumor [7]. Tumor-infiltrating lymphocytes and a marked inflammatory response are associated with host response. That may result in local tumor growth control and even complete regression of tumor; as in some head and neck tumors [7] and malignant melanoma [12,13].

2. Primary tumors of the tonsil that present cystic neck metastasis are said to be associated with a slower growth rate than tumors that produce solid metastases [2]. The indolent growth of the primary tumor can be explained by the intimate functional relationship of lymphocytes with crypt lymphoepithelium. That explains the apparently slow-growing, indolent nature of many crypt epithelial carcinomas [2].

Similarly, lymphocytes have influence on metastatic tumor cells and malignant cells could have lower growth rate in lymph nodes than in primary tumor site due to the presence of noticeable immunologic response. This lower growth rate could also be related to the fact that lymph nodes like many other metastatic sites, in contrast to the primary site, may be harsh environments for tumor cells to grow [14].

To conclude, frequency of cystic structures in metastatic lymph nodes rather than primary sites is due to the more silent presentation of tumor and expression of parental properties.

### Distant metastatic locations and cyst formation

Reports of cystic features in few distant metastatic locations from oro/nasopharyngeal SCC to lung, skin and bone may speak against the previous statement that implies the lymphatic environment induces cyst formation. Indeed, cyst formation is the inherent property of tumor cells, which is maintained and expressed in the permissive environment of lymphoid tissue. This suitable environment could rarely be present in other metastatic sites.

### Genetic content of tumor in primary and metastatic lymph nodes

Genetic content of primary and metastatic tumors have been compared in some investigations. Barker et al [15] showed that each cancer within the head and neck maintained its expression profile between primary site and nodal metastases. Sasatomi et al [14] compared accumulated allele loss between primary tumor and lymph node metastases in non-small cell lung carcinoma and found that the maximally accumulated load of mutational change in lymph node metastases was significantly smaller than that of primary tumor. This event could be explained by the fact that metastatic spread of malignant cells occurs early and before more mutations are acquired [14]. Although similar investigations are not present for other tumors, the results of this study could be true in other malignancies including head and neck squamous cell carcinoma. Therefore, metastatic cells with less mutational change are genetically more similar to their parent cells and have more probability for cyst formation.

Finally, more investigations on heterogeneity between the primary and metastatic tumor could help to understand mechanisms of metastasis in head and neck cancers [16], the way that hopefully will end in recognition of cancer progression completely.

## Conclusion

Some squamous cell carcinomas (SCCs) have cystic metastases in lymph nodes. Majority of these cases are pseudocysts. However, some are true cystic cavities. There are strong evidences that show some of SCCs with true cystic metastases originate from salivary ducts. The other probable mechanism of real cyst formation is that these tumors are cancers of transitional type rather than a usual SCC. They originate from transformed keratinocytes and have intrinsic property for cyst formation. On the other hand, strong immunologic host response is present in lymph nodes; so malignant cells of lymph nodes have lower proliferation rate. Moreover, metastatic cells have less mutational change than primary tumor cells. These can lead to the silent presentation of tumor and expression of parental properties in metastatic lymph nodes.

## List of abbreviations

SCC: Squamous Cell Carcinoma; HPV: Human Papilloma Virus; CK7: Cytokeratin7.

## Competing interests

The author declares that she has no competing interests.
